# Prenatal Androgenization Modifies H3K9me3 Binding in the Promoter of the Androgen Receptor Gene in the Arcuate Nucleus of the Adult Female Mouse

**DOI:** 10.1007/s43032-026-02174-9

**Published:** 2026-08-21

**Authors:** Yugo Watanabe, Kelly A. Glendining, Lorryn Fisher, Rebecca E. Campbell, Christine L. Jasoni

**Affiliations:** 1 Centre for Neuroendocrinology, Department of Anatomy, School of Biomedical Sciences, University of Otago, Dunedin, New Zealand; 2 Department of Biochemistry, School of Medicine, Asahikawa Medical University, Asahikawa, Japan; 3 Centre for Neuroendocrinology, Department of Physiology, School of Biomedical Sciences, University of Otago, Dunedin, New Zealand; 4 Faculty of Agriculture and Life Sciences, Lincoln University, Lincoln, New Zealand

**Keywords:** Polycystic ovary syndrome, Androgen receptor, Prenatal androgen exposure, Arcuate nucleus, Histone modification, H3K9me3

## Abstract

Animal models have shown that prenatal exposure to excess androgens is associated with the development of polycystic ovary syndrome (PCOS) features. We have identified that prenatally androgenized (PNA) mice modelling PCOS show suppressed androgen receptor mRNA (*Ar*) expression in the arcuate nucleus (ARC) across development. This could contribute to PCOS-related impaired gonadal steroid hormone feedback to GnRH neurons. However, the mechanism of *Ar* mRNA suppression following PNA is not determined. We performed a chromatin immunoprecipitation (ChIP) assay coupled with quantitative PCR (qPCR) to investigate histone and transcription factor binding within the *Ar* gene promoter or enhancer regions in the ARC of female mice at postnatal day (P)60 or gestational day (GD)18.5. In comparison to adult vehicle control (VEH) mice, our ChIP-qPCR revealed that H3K9me3, a repressive histone mark, was increased in adult PNA mice at the promoter regions of the *Ar* gene. H3K27ac, an active histone mark, and SP1, a transcription factor that acts as a positive regulator of gene expression, were unchanged in the same regions. Increased H3K9me3 seen at the promoter region in adult PNA mice, however, was not observed in the GD18.5 mice ARC following PNA. These results suggest that the deposition of H3K9me3 on the *Ar* promoter is unlikely to be established by PNA at the time of excess androgen exposure, and instead is likely to be established later in postnatal development or in adulthood. These data provide greater understanding of the developmental mechanisms and timeline underpinning PNA-mediated female dysfunction and PCOS-like reproductive physiology.

**Supplementary Information:**

The online version contains supplementary material available at https://doi.org/10.1007/s43032-026-02174-9.

## Introduction

Animal and human research suggests that prenatal androgen exposure contributes to the development of polycystic ovary syndrome (PCOS), an endocrine disorder that causes anovulatory infertility [1, 2]. Although the precise etiology of PCOS remains unclear, androgen-induced impairments in the responsiveness of the brain to gonadal steroid hormone feedback is implicated. Gonadal hormone negative feedback, acting via steroid hormone-sensitive afferent neurons in the arcuate nucleus of the hypothalamus (ARC), is critical for the appropriate regulation of the reproductive axis [3]. Studies using a prenatally androgenized (PNA) mouse model of PCOS have shown elevated circulating androgens and impaired gonadal hormone feedback to GnRH neurons [4–6]. This adult phenotype is associated with suppressed mRNA expression of the androgen receptor (*Ar*), a nuclear receptor that mediates the cellular actions of androgens, in the ARC of the PNA-induced PCOS mouse model [7]. This suppression of *Ar* is long-term, spanning development stages from GD17.5 through to adulthood, and could potentially contribute to impaired gonadal hormone negative feedback.

Although the mechanism of *Ar* mRNA suppression following PNA is not yet determined, histone modifications may be a particularly important post-translational modification (PTM) for regulation of *Ar.* Previous study in cultured human-derived fibroblasts has shown that repression of *AR* is associated with an increase in the repressive histone mark, histone 3 lysine 9 trimethylation (H3K9me3), and a decrease in the active histone mark, histone 3 lysine 27 acetylation (H3K27ac), at the *AR* promoter region [8]. In a sheep model of PCOS treated with prenatal testosterone, protein level of H3K9me3 is incrased on promoters of downgulated genes, while ptotein level of H3K27ac is decrased on promoters of upregulated genes [9]. In addition, transcription factors and histone modifications often cooperate at regulatory elements to regulate the expression of target genes [10]. For instance, SP1 is a transcription factor that acts as a positive regulator of gene expression by binding to GC-box elements, and is strongly enriched in promoter or enhancer regions [11, 12]. Growing evidence suggests that the epigenome exhibits a high degree of plasticity during development and can be modulated by external factors in the uterine environment [13, 14]. Therefore, we hypothesized that the suppression of *Ar* in the ARC of PNA-induced PCOS mice is associated with H3K9me3 and/or H3K27ac modifications in the *Ar* gene promoter or enhancer regions. As the co-localization of transcription factors with histone modifications is crucial for understanding how these regulators control gene expression, we also investigated the level of SP1 binding to the *Ar* gene promoter region in the ARC of adult female mice following PNA.

In this study, we investigated the enrichments of H3K9me3 and H3K27ac on the putative *Ar* gene promoter and enhancer regions in the ARC of PNA or vehicle control (VEH) adult female mice. Additionally, to clarify whether the histone modifications observed at the adult stage are formed immediately after the PNA treatment, we assessed the enrichment of H3K9me3 in the *Ar* gene promoter and enhancer regions in the ARC of gestational (GD) 18.5 female mice following PNA. Lastly, we explored the enrichment of the SP1 transcription factor in the putative *Ar* promoter region in the ARC of adult PNA or VEH mice.

## Methods

### Animals

Adult male and freely cycling adult female mice between 60 and 80 days old were housed under 12-h light cycles with food and water available at ad libitum. PNA treatment to model PCOS was carried out as previously described [4–7]. Breifly, on GD15.5–17.5 (with day of vaginal plug defined as GD 0.5), pregnant dams were given daily s.c. injections of sesame oil (100 μl) either as a vehicle control (VEH group) or containing 250 μg dihydrotestosterone (DHT, PNA group). PNA- and VEH-treated female offspring were then studied.

### Tissue Preparation for ChIP-qPCR

**Fig. 1:**
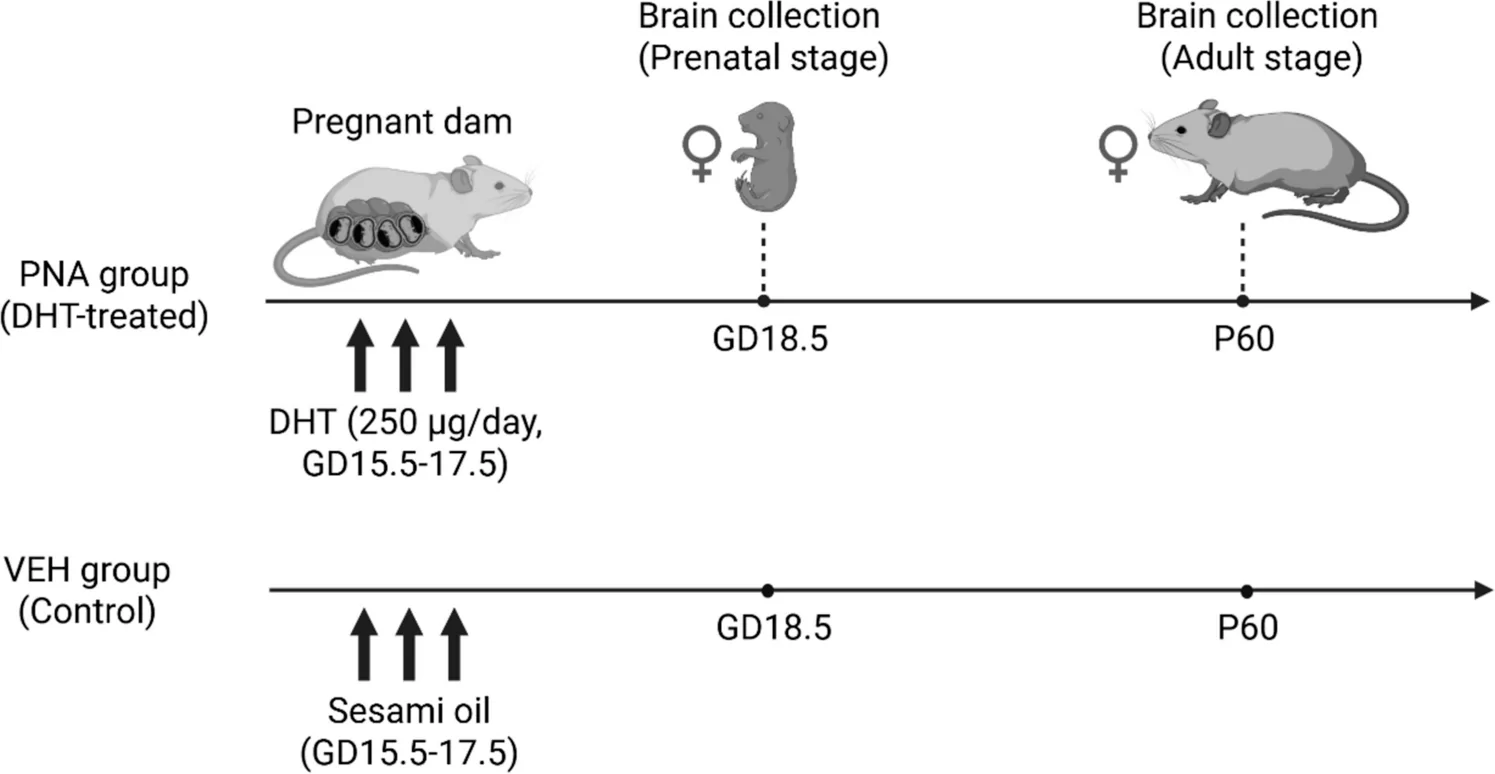
Experimental design and timeline of the prenatal androgenization (PNA) model. Pregnant dams were treated with DHT (250 μg/day, GD15.5–17.5) to induce prenatal androgen exposure. Brains were collected from PNA-treated female offspring at GD18.5 (prenatal stage; 24 h after the final DHT treatment) or P60 (adult stage). Vehicle controls (VEH) underwent the same procedure. Figure is created with BioRender.com

Brains were collected from VEH- or PNA-treated female offspring at P60 or GD18.5 (Fig. 1). P60 female mice were sacrificed by cervical dislocation, in the diestrus stage of the estrous cycle as determined by vaginal cytology. Protocols in Han et al. [15] and Salehi et al. [16] were used as references to extract the ARC tissues from fresh mouse brains. Once the isolated brain was placed in the mouse brain matrix (Argotech, Dunedin, New Zealand), a chilled razor blade was placed through the optic chiasm and another blade was placed through the mammillary body at 1 mm interval. The sections containing ARC and median eminence were excised by cutting approximately 0.5 mm to each lateral side of the median eminence under a dissection microscope as previously described by our study [7]. For GD 18.5 brains, timed-pregnant VEH and PNA dams were sacrificed by cervical dislocation 24 h after the final daily s.c. injections. Embryos were then collected by caesarean section and decapitated. Sex of the GD18.5 mice was determined by gonad morphology [17], and female fetuses were collected from each pregnancy. The ARC was microdissected under a dissection microscope. The microdissected ARC from P60 or GD18.5 brains were then crosslinked in 1% formaldehyde for 10 min at room temperature. Formaldehyde was then quenched with glycine at a final concentration of 13 mM, and tissue was stored at − 80 °C until required.

### ChIP-qPCR

ChIP was performed using the ChIP-validated rabbit polyclonal antibodies; anti-H3K9me3 (Abcam, ab8898, 2 μg), anti-H3K27ac (Abcam, ab4729, 4 μg) or anti-SP1 (proteintech, 21,962–1-AP, 4 μg). Rabbit IgG (Abcam, ab171870, 2 or 4 μg) was used for an isotype control. These antibodies were conjugated to protein G Dynabeads (ThermoFisher Scientific) in 0.5% bovine serum albumin (BSA) for 3 h at 4 °C. Fixed tissues were homogenized and lysed in 1 × Halt™ Protease Inhibitor Cocktail (ThermoFisher Scientific), followed by chromatin shearing using Micrococcal Nuclease (Life Technologies, used 300 U for H3K9me3 and H3K27ac ChIP, and 50 U for SP1 ChIP) for 20 min at 37 °C. The extracted chromatin was then immunoprecipitated with the Dynabeads-conjugated antibody overnight at 4 °C. Reverse crosslinking was performed for 4 h at 65 °C, followed by additional incubation with Proteinase K (A&A Biotechnology) for 2 h at 55 °C. DNA was purified with QIAquick PCR Purification Kit (QIAGEN), and eluted in 30 μl nuclease-free water. All qPCR assays were performed on the Viia7 Real-Time PCR System (Applied Biosystems). Triplicate (10 μL) reactions contained 2 × PowerUp SYBR Green, 1 μL DNA and 250 nM forward and reverse primers. Each qPCR run included no-template control as negative control. Histone binding of H3K9me3, H3K27ac, or SP1 binding were assessed at a putative promoter region or two separate enhancer regions of the *Ar* gene as defined by the UCSC Genome Browser (https://genome.ucsc.edu). ChIP-qPCR primers were designed using PrimerBLAST (http://www.ncbi.nlm.nih.gov/tools/primerblast), or obtained from previously published work (Table 1). ChIP-qPCR analysis of P60 tissue was derived from n = 4–6 independent pregnancies. For GD18.5, tissue was derived from n = 6–8 independent pregnancies, and for each n, 3–4 female fetuses within a litter were pooled. ChIP-qPCR data were normalized to input chromatin using the Delta Ct method.

**Table 1:** Primers used for ChIP-qPCR analysis

Gene	Forward (F) or reverse (R)	Primer sequences (5’–3’)	Amplicon length (bp)	GenBank accession no. and references
*Ar −204* to −134	F	ACAGTGAGTCATTCCAGCCTTCA	70	NC_000086.8
	R	GCTAGCGCAGAGTGGCTTTAGA		
*Ar* −17 to + 54	F	TTTGCTTTCCACCTCCCAGCG	71	NC_000086.8
	R	GGACCCTCTGGTTCTCCCAG		
*Ar* + 1420 to + 1489	F	AGATCAGGATGACTCAGCTGCC	70	NC_000086.8
	R	GCTGCTTAAGCCTGGGAAAGTG		
*Ar* + 2147 to + 2235	F	CATCCACACGCCCGTATCAA	89	NC_000086.8
	R	AAGTCCCCATAGCGGCATTG		
*IntReg1*	F	CCGTGCGAGGTTCTGGATCT	84	NC_000067.7
	R	CTGCCCAGATGCTCCAAAGC		
*IntReg2*	F	GTGTTGCTGGCTCATGTAGGA	150	NC_000086.8
	R	TCAAGAACAGAGCACTGACCCAA		
*IntReg3*	F	CATCCAGGGACGTGCTGACT	95	NC_000072.7 (Fukuda et al. [25])
	R	TGTGTTCTCCCCTCACTGATCTC		

### *In silico* Analysis of Putative *cis*-regulatory Elements for SP1

Putative *cis*-regulatory elements of SP1 in mouse *Ar* promoter were searched using the online transcription factor binding profile database, JASPAR (http://jaspar.genereg.net). Selected promoter sequences were scanned with 80% (default setting) relative profile score threshold.

### Statistics

Data were analyzed with Prism version 9.5.1. Normality of all the data was determined by the Shapiro–Wilk test, and variance equality was determined by Spearman’s test or F-test. The enrichment of H3K9me3, H3K27ac or SP1 between PNA or VEH animals was determined by unpaired Student’s t-tests. Results were considered statistically significant where *P* < 0.05.

## Results

**Fig. 2:**
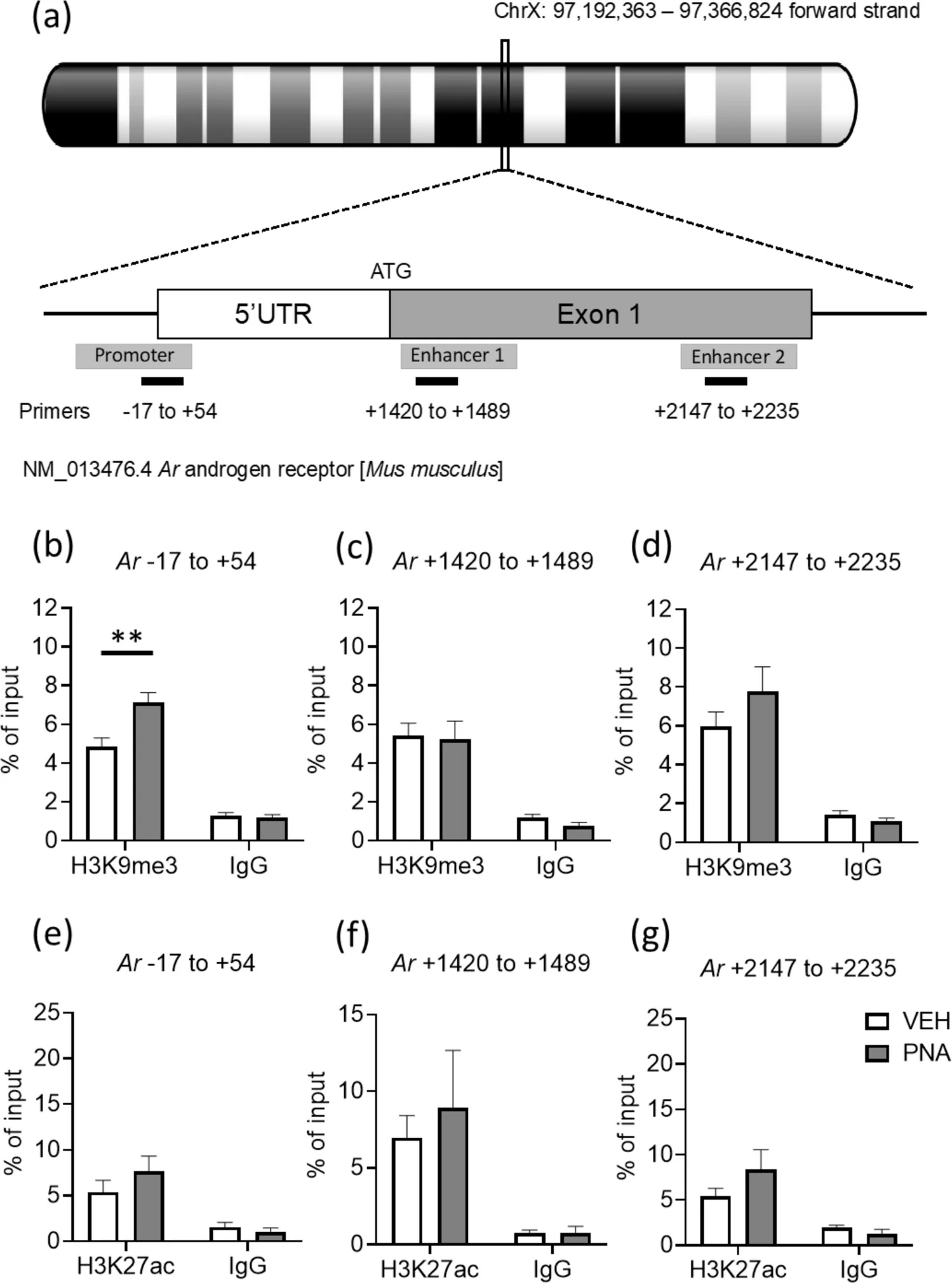
ChIP-qPCR analysis of H3K9me3 or H3K27ac binding in the putative *Ar* promoter, or within two enhancer regions in the ARC of adult VEH or PNA mice. Primer target sites in the *Ar* promoter or enhancer regions were detected by UCSC Genome Browser (**a**). Bar graphs show enrichment of H3K9me3 and IgG (negative control) in the target sites in the *Ar* promoter (**b**) −17 to + 54, or enhancer regions, (**c**) + 1420 to + 1489 bp or (**d**) + 2147 to + 2235. Bar graphs show enrichment of H3K27ac and IgG in the target sites in the *Ar* promoter (**e**) −17 to + 54, or enhancer regions, (**f**) + 1420 to + 1489 bp or (**g**) + 2147 to + 2235. Data shown are means ± SEM (n = 4–6). Asterisks indicate significant difference in % of input between VEH and PNA **P < 0.01 (unpaired t-test)

We first investigated the enrichment of H3K9me3 or H3K27ac in the putative *Ar* promoter region and two enhancer regions in the ARC of adult VEH control or PNA mice (n = 4–6, Fig. 2a). In the *Ar* promoter region at −17 to + 54 from transcription start site (TSS), significantly higher levels of H3K9me3 binding were found in PNA mice (7.12 ± 0.53%) compared to VEH mice (4.84 ± 0.46%, t = 3.28, *df* = 9, P = 0.0096, Fig. 2b). In the *Ar* enhancer region at + 1420 to + 1489, there was no significant difference in PNA mice (5.24 ± 0.93%) compared to VEH mice (5.41 ± 0.65%, t = 0.16, *df* = 8, P = 0.88, Fig. 2c). In the *Ar* enhancer region at + 2147 to + 2235, there was no significant difference in PNA mice (5.98 ± 0.74%) compared to VEH mice (7.75 ± 1.2%, t = 1.2, *df* = 10, P = 0.26, Fig. 2d). In the promoter region at −17 to + 54 from TSS, no significant difference in the level of H3K27ac binding was observed in PNA mice (7.66 ± 1.66%) compared to VEH mice (5.34 ± 1.29%, t = 1.08, *df* = 10, P = 0.30, Fig. 2e). In the *Ar* enhancer region at + 1420 to + 1489, there was no significant difference in PNA mice (8.90 ± 3.75%) compared to VEH mice (6.96 ± 1.45%, t = 0.52, *df* = 9, P = 0.61, Fig. 2f). In the *Ar* enhancer region at + 2147 to + 2235, there was no significant difference in PNA mice (8.41 ± 2.15%, *n* = 6) compared to VEH mice (5.42 ± 0.85% t = 1.30, *df* = 10, P = 0.22, Fig. 2g). The enrichment of H3K9me3 and H3K27ac was low when primers specific to an intergenic region in chromosome 1 were used as negative control (Supplemental Fig. 1).

**Fig. 3:**
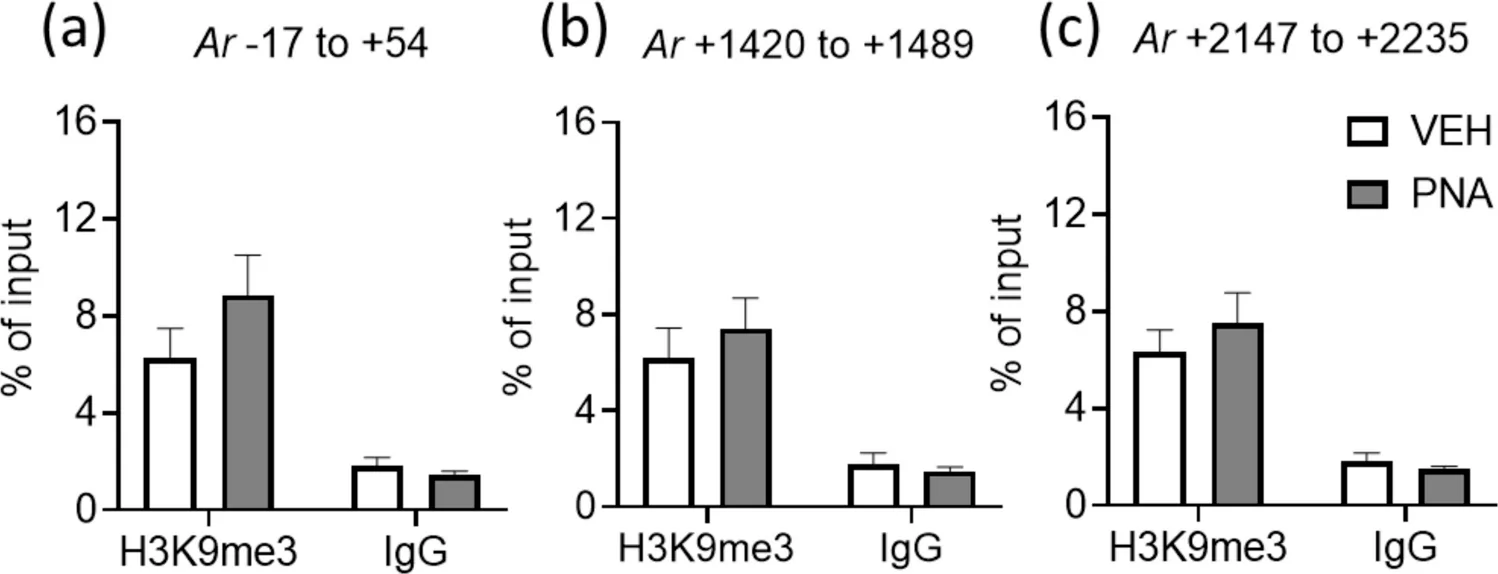
ChIP-qPCR analysis of H3K9me3 binding in the putative *Ar* promoter and within two enhancer regions in the ARC of GD18.5 VEH or PNA mice. Bar graphs show enrichment of H3K9me3 and IgG (negative control) at the target sites in the *Ar* promoter (**a**) −17 to + 54, or enhancer regions, (**b**) + 1420 to + 1489 bp or (**c**) + 2147 to + 2235. Data shown are means ± SEM (n = 6–8)

We next investigated the enrichment of H3K9me3 in the putative *Ar* promoter and within two enhancer regions of ARC tissue collected from GD18.5 VEH or PNA mice (n = 6–8, Fig. 3). In the *Ar* promoter region at −17 to + 54 from TSS, no significant difference in the level of H3K9me3 binding was observed in PNA ARC (8.87 ± 1.64%) compared to VEH mice (6.28 ± 1.21%, t = 1.231, *df* = 11, P = 0.24, Fig. 3a). In the *Ar* enhancer region at + 1420 to + 1489, there was no significant difference in PNA ARC (7.41 ± 1.27%) compared to VEH ARC (6.19 ± 1.25%, t = 0.69, *df* = 10, P = 0.51, Fig. 3b). In the *Ar* enhancer region at + 2147 to + 2235, there was no significant difference in PNA ARC (7.52 ± 1.25%) compared to VEH ARC (6.31 ± 0.94% t = 0.73, *df* = 12, P = 0.48, Fig. 3c). The enrichment of H3K9me3 was low when primers specific to intergenic region in chromosome 6 were used as negative control (Supplemental Fig. 1).

**Fig. 4:**
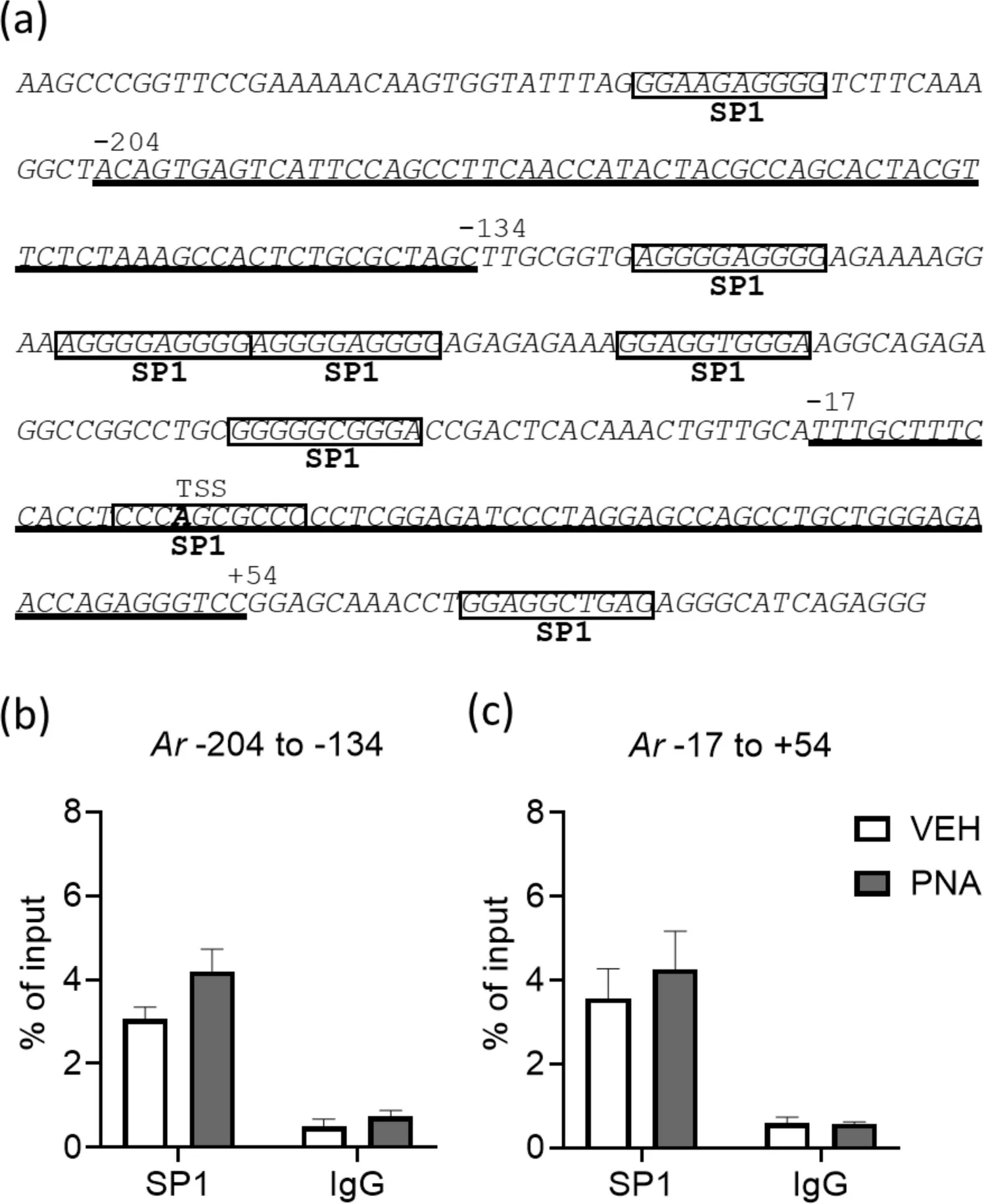
ChIP-qPCR analysis of SP1 binding in the putative *Ar* promoter region in the ARC of adult VEH or PNA mice. (**a**) Putative *cis*-regulatory elements of SP1 in the promoter region of mouse *Ar* detected by JASPAR. Sequences targeted by qPCR primers are underlined. Numbers indicate the position of the target sequences from the *Ar* transcription start site. Bar graphs show enrichment of SP1 and IgG (negative control) in the target sites within the *Ar* promoter (**b**) −204 to −134 or (**c**) −17 to + 54 bp. Data shown are means ± SEM (n = 5–6). *TSS*, transcription start site

Lastly, we performed a JASPAR analysis on the putative *Ar* promoter region for the SP1 transcription factor. Eight potential SP1 binding sites were detected at a relative profile score threshold of 80% by analyzing the putative promoter region of *Ar* (Fig. 4a). The enrichment of SP1 in the putative *Ar* promoter region was investigated in the ARC of adult VEH or PNA mice. In the *Ar* promoter region at −204 to −134 from TSS, there was no significant difference in PNA ARC (4.20 ± 0.53%) compared to VEH ARC (3.06 ± 0.29%, t = 1.99, *df* = 9, P = 0.08, Fig. 4b). In the promoter region at −17 to + 54 from TSS, no significant difference in the level of SP1 was observed in PNA ARC (4.26 ± 1.00%) compared to VEH ARC (3.56 ± 0.72%, t = 0.60, *df* = 10, P = 0.56, Fig. 4c). The enrichment of SP1 was low when primers specific to an intergenic region in chromosome X were used as negative control (Supplemental Fig. 1).

## Discussion

We conducted ChIP coupled with qPCR to investigate whether altered histone modifications within the *Ar* gene promoter or enhancer regions may underpin decreased *Ar* mRNA expression in the ARC of PNA mice across development. In comparison to adult VEH mice, our ChIP-qPCR revealed an increased level of H3K9me3 in adult PNA ARC at the putative promoter region of *Ar* gene, while the levels of H3K27ac and SP1 remained unchanged. This suggests that the heterochromatin marked with repressive histone mark H3K9me3 at the *Ar* promoter region could be a contributing factor to the suppression of *Ar* expression observed in adult PNA ARC. The observed change in H3K9me3 at the *Ar* promoter region was not evident in the GD18.5 mice ARC directly following PNA. These results suggest that the accumulation of H3K9me3 at the *Ar* promoter is not established at the time of excess androgen exposure, and is instead likely to be established later in postnatal development, or in adulthood as a consequence of events that arise as a result of PNA exposure in utero.

In our previous study, using GD17.5, P0, P25, P40 and P60 female mice, we found that *Ar* mRNA levels in the ARC of PNA mice were significantly decreased compared to VEH mice across all ages [7]. The observed increase in H3K9me3 in the *Ar* promoter at P60 is consistent with current understanding of the function of H3K9me3 at gene promoters, which typically correlates with transcriptional repression [18, 19]. Previous studies have demonstrated that histone modifications play a key role in impairing the expression of several genes in PCOS models. For instance, the expression of the aromatase gene *CYP19A1* in human ovarian cumulus cells is higher in PCOS patients compared to controls and correlates with hypomethylation of H3K9 on the promoter [20]. Moreover, a study in adult ovaries of a prenatal testosterone-treated PCOS model identified upregulated and downregulated genes important for ovarian function and those gene expression changes were correlated with histone modifications on the promoters, including H3K9me3 [9]. These results suggest that histone modifications could be altered on some key genes as part of the etiology of PCOS, possibly by consequence of early developmental programming by PNA, or high circulating testosterone postnatally. The timing of when the increased H3K9me3 is formed on the *Ar* promoter in PCOS is currently not known.

Our study revealed that neither the promoter nor enhancer regions of *Ar* showed increased of H3K9me3 in the ARC of GD18.5 PNA mice compared to VEH controls. The observation that GD18.5 mice lack the modification, may suggest that the observed histone modification is not a direct consequence of PNA exposure, but instead occurs through later developmental events dependent on PNA exposure. While the addition of a single PTM can function to positively or negatively affect gene transcription, there is also cross-talk between histone modifications, such that a modification not screened in this study may be altered at the time of PNA to influence H3K9me3 binding in ARC in adulthood. Whether the protein level of H3K9me3 is specifically upregulated in the adult stage in the ARC of PNA mice is currently not known. An extensive examination of protein levels of H3K9me3 and histone status of the *Ar* gene across development and into adulthood would provide clarity in this regard.

Contrary to our hypothesis, our present study showed no changes in the occupancy of SP1 on the *Ar* promoter region in the ARC of adult PNA females compared to VEH controls. This result suggests that the accessibility of other positive regulators is reduced by the occupancy of H3K9me3 on the *Ar* promoter. Conversely, in relation to the H3K9me3 increase, transcription factors that function as negative regulators could be involved in maintaining the repressive histone status, thereby causing the suppression of *Ar* transcription. For instance, ZFP57, which binds to mammalian CpG site, is shown to be necessary for the maintenance of the H3K9me3 mark and DNA methylation at the CpG sites. Moreover, studies on human *AR* reported that negative regulators, such as c-Jun and p53, which bind to the *AR* promoter region, are associated with the transcriptional repression of the *AR* in androgen-sensitive human prostate adenocarcinoma cells [21–23]. Further study is required to investigate the binding of negative regulators on the *Ar* promoter that takes place in the repression of *Ar*.

The present study has some limitations. Although we microdissected the ARC pieces using an approach compaired to previous ChIP studies [15, 24], it was challenging to completely eliminate the presence of non-ARC cells from adjacent brain regions, and their chromatin may have contributed to the pool on which ChIP-qPCR was performed. Secondly, although positive binding of SP1 was detected with the primer sets targeting sequences within the promoter region, the ChIP-qPCR primers generated did not include all of the potential SP1 binding sites detected by JASPR due to constraints in primer design options. Alternative techniques that do not require target specification with primers, such as ChIP-seq, would be advantageous for future study.

## Conclusion

The increase in H3K9me3 occupancy on the *Ar* promoter in the adult PNA ARC provides support for one potential mechanism for the suppression of *Ar* expression that we previously reported in PNA mice [7]. Further examination of the H3K9me3 status across postnatal development in PNA and controls would clarify the temporal onset of H3K9me3 occupancy on the *Ar* gene. The landscape of transcriptional modulators that bind directly and indirectly to DNA at regulatory regions is exhaustive, so considerable further examination is required to understand the full extent of factors that influence regulation of *Ar* transcription.

## Supplementary Information

Below is the link to the electronic supplementary material.

Supplementary file1 (DOCX 40 KB)

## Data Availability

The original contributions presented in the study are included in the article and　supplementary materials. Further inquiries can be directed to the corresponding author.

## Abbreviations

AR/Ar: Androgen receptor
ARC: Arcuate nucleus
GnRH: Gonadotropin-releasing hormone
H3K27ac: Histone 3 lysine 27 acetylation
H3K9me3: Histone 3 lysine 9 trimethylation
LH: Luteinizing hormone
PCOS: Polycystic ovary syndrome
PNA: Prenatal androgen exposure
