# Supplementary material for: Prenatal Androgenization Modifies H3K9me3 Binding in the Promoter of the Androgen Receptor Gene in the Arcuate Nucleus of the Adult Female Mouse
Source: Reprod Sci. 2026 Aug 21;33(9):1775–82. doi: 10.1007/s43032-026-02174-9 (PMC13562324; doi:10.1007/s43032-026-02174-9)
Supplement: Supplementary file 1 [file 43032_2026_2174_MOESM1_ESM.docx]

**Prenatal androgenization modifies H3K9me3 binding in the promoter of the androgen receptor gene in the arcuate nucleus of the adult female mouse**

**Reproductive Sciences**

**Yugo Watanabe*^1,2^, Kelly A. Glendining^3^, Lorryn Fisher^1^, Rebecca E. Campbell^3^, Christine L. Jasoni^1,4^**

^1^Centre for Neuroendocrinology, Department of Anatomy, University of Otago, School of Biomedical Sciences, Dunedin, New Zealand

^2^Department of Biochemistry, School of Medicine, Asahikawa Medical University, Asahikawa, Japan

^3^Centre for Neuroendocrinology, Department of Physiology, University of Otago, School of Biomedical Sciences, Dunedin, New Zealand

^4^Faculty of Agriculture and Life Sciences, Lincoln University, Lincoln, New Zealand

*** Correspondence:**

Yugo Watanabe
[yugo_watanabe](mailto:yugo.watanabe@otago.ac.nz)@asahikawa-med.ac.jp

**Supplemental data**

**Fig. S1**. ChIP-qPCR analysis of H3K9me3, H3K27ac or SP1 binding in the ARC of adult or GD18.5 VEH or PNA mice targeting intergenic regions as negative controls. Bar graphs show enrichment of (a) H3K9me3 and IgG in the adult ARC, (b) H3K27ac and IgG in the adult ARC, (c) SP1 and IgG in the adult ARC, (d) H3K9me3 and IgG in the GD18.5 ARC in the selected intergenic regions. Data shown are means ± SEM (n=5-8). Abbreviation: IntReg, intergenic region.
